# Neighborhood Influences on Late Life Cognition in the ACTIVE Study

**DOI:** 10.1155/2012/435826

**Published:** 2012-08-26

**Authors:** Shannon M. Sisco, Michael Marsiske

**Affiliations:** Department of Clinical and Health Psychology, University of Florida, Gainesville, FL 32610, USA

## Abstract

Low neighborhood-level socioeconomic status has been associated with poorer health, reduced physical activity, increased psychological stress, and less neighborhood-based social support. These outcomes are correlates of late life cognition, but few studies have specifically investigated the neighborhood as a unique source of explanatory variance in cognitive aging. This study supplemented baseline cognitive data from the ACTIVE (Advanced Cognitive Training for Independent and Vital Elderly) study with neighborhood-level data to investigate (1) whether neighborhood socioeconomic position (SEP) predicts cognitive level, and if so, whether it differentially predicts performance in general and specific domains of cognition and (2) whether neighborhood SEP predicts differences in response to short-term cognitive intervention for memory, reasoning, or processing speed. Neighborhood SEP positively predicted vocabulary, but did not predict other general or specific measures of cognitive level, and did not predict individual differences in response to cognitive intervention.

## 1. Introduction

The neighborhood has emerged as a prominent level of analysis in studies of contextual influences on health and wellbeing in human development. This owes to the social organization of life within neighborhoods, and to the availability of data at neighborhood-like levels of analysis (i.e., census tracts). Neighborhood context has also received greater attention due to ecological psychology's conceptualization of human development as the product of a dynamic interaction between the individual and multiple nested environments, of which the neighborhood is one of the most immediate environments [1]. Contemporary scholarship on neighborhood factors has also highlighted the importance of understanding whether neighborhood variables add explanatory variance above and beyond individual differences. 

Neighborhood socioeconomic status (SES) is the most consistently reported neighborhood-level predictor of cognitive outcomes—certainly in childhood, and potentially across the life span [2]. SES is also the strongest and most consistently reported neighborhood-level predictor of health outcomes among older adults—a relationship which may have a cumulative effect across the life span [3]. The association of neighborhood-level SES with health is in fact stronger among adults aged 60–69 than among young and middle-aged adults, and during these years its association with health is comparable to or stronger than the relationship of individual SES to health [4]. In addition to predicting poorer physical health, lower neighborhood SES is also associated with reduced rates of physical activity, increased incidence of depression and psychosocial stress, and less neighborhood-based social support and social engagement [3]. Many of these outcomes have also been identified as correlates of late life cognition [5], yet few studies to date have specifically investigated the role of the neighborhood in cognitive aging. 

While neighborhood effects on late life cognition have received less attention, neighborhood effects on early-life cognitive development are relatively well documented. Neighborhood SES is positively correlated with cognitive development across the entire spectrum of child development, from the prenatal stages (in the form of reduced rates of congenital anomalies and neural tube defects among higher-SES neighborhoods, presumably due to reduced risk of exposure to environmental toxins; [6, 7]) and preschool years [8, 9] to academic achievement in the school age [10, 11] adolescent, and young adulthood years (e.g., high school graduation rates and college attendance [12]). 

Four recent studies explored relationships between late life cognition and the neighborhood context on a broad scale. Wight and colleagues [13] found that after controlling for individual-level education and area-level median household income, elders living in areas with low neighborhood-level educational attainment (defined by census tract area) achieved lower cognitive status scores compared to elders living in areas with high neighborhood-level educational attainment (as assessed using the mini-mental state examination or MMSE [14]). A United Kingdom study [15] reported a clear and significant downward trend in cognitive performance on the MMSE and tests of verbal fluency and memory for older individuals living in neighborhoods with greater deprivation, after controlling for individual-level wealth, income, and education, across all age and gender groups. These effects were also robust after adjusting for the effects of individual systolic blood pressure, history of stroke, and duration of residence in the current neighborhood. Sheffield and Peek [16] moved beyond cross-sectional analysis to examine how neighborhood SES and ethnic composition (proportion Mexican American) predicted 5-year change in MMSE score in a US national sample. Independent of individual-level risk factors, odds and rate of incident cognitive decline increased as a function of lower neighborhood SES and decreased with the proportion of Mexican American neighborhood residents. Finally, recent findings from the Baltimore Memory Study suggest that the relationship between neighborhood and late life cognition may also depend on genotype [17]. Specifically, neighborhood psychosocial hazards were not found to be related worse cognitive performance. However, apolipoprotein E4 genotype was found to interact with high neighborhood psychosocial hazards, resulting in poorer cognitive performance on measures of processing speed and executive functioning after controlling for individual-level covariates. This suggests evidence of a gene-environment interaction in neighborhood's relation to late life cognition.

The existing studies focused on broad differences in late life cognitive *status *(i.e., MMSE score), but did not investigate the effect of neighborhood on different cognitive domains (see recommendations by Cullum et al. [18]). Furthermore, the use of the MMSE in these studies was not an ideal measure of cognition, as they targeted samples of initially healthy, independent elders. The MMSE is not sensitive to differences among healthy older adults, having been designed to screen for dementia [14]. Cognitive aging research has instead focused increasingly on the use of confirmatory factor analysis (CFA) and structural equation modeling (SEM) to explore whether differences in late life cognitive performance are related to general or specific processes. It has also been noted that conceptualizing late life cognition using a single general factor (“*g*”) is a simplistic approach to a complex and dynamic cognitive system [19]. This suggests neighborhood influences should be investigated at both general and domain-specific levels. Neighborhood effects may support some dimensions of cognition more strongly than others, in addition to influencing general cognitive status. 

The present study aimed to identify whether neighborhood effects on cognition were global, or specifically related to particular cognitive domains such as memory, reasoning, processing speed, everyday cognition, or vocabulary. Another innovation of the present study is the capacity to address whether neighborhood effects might also influence the magnitude of potential benefit from cognitive intervention. No study, in part because of a lack of data availability, has examined this question. Elucidating both the specificity of neighborhood effects on particular domains of cognition and the extent to which neighborhood affects cognitive training response may provide additional hints toward potential mechanisms by which the neighborhood exerts influence. For example, effects on the fluid cognitive abilities such as processing speed, memory, and reasoning (and their response to cognitive training) would suggest a more biologically mediated influence given their sensitivity to brain integrity. In contrast, effects on measures of crystallized cognitive abilities, especially vocabulary, might hint toward a more socioculturally mediated, and possibly lifespan accumulative influence, as crystallized cognitive abilities are developed with the accumulation of verbal knowledge, developed over a lifetime of experience in engaging with culture [20]. As this lifelong accumulation is strongly influenced during childhood, adolescence, and young adulthood, effects of current neighborhood SEP on crystallized abilities may also reflect, in part, effects of early-life neighborhood SEP.

The Advanced Cognitive Training for Independent and Vital Elderly (ACTIVE) study was a randomized, controlled clinical trial examining the effects of three cognitive interventions for community-dwelling older adults [21]. In ACTIVE, older adults from six US catchment areas (Baltimore/coastal Maryland; the metropolitan areas of Birmingham, Boston, Detroit, and Indianapolis; central Pennsylvania) completed a baseline assessment including multiple cognitive measures. Participants were then randomized to one of three ten-session cognitive intervention programs or a no-contact control condition. 

The present study sought to examine whether neighborhood socioeconomic position (SEP) shows bivariate relationships with baseline cognitive level and immediate response to cognitive training, beyond individual-level predictors of cognition. If so, we aimed to discern whether neighborhood effects were significant for general cognitive ability (“*g*”), and whether effects were differential for specific cognitive abilities (memory, reasoning, speed, everyday cognition, and vocabulary). We hypothesized that the potential effect of neighborhood SEP may most likely occur by way of sociocultural processes, and therefore would have significant positive associations with general cognitive ability and with crystallized cognitive domains, including vocabulary. Neighborhood SEP was hypothesized to have relatively weaker, if detectable, associations with fluid cognitive domains, including memory, reasoning, speed, and everyday cognition.

## 2. Method

### 2.1. Study Design

The primary objective of ACTIVE was to test the effectiveness and durability of three cognitive interventions. The initial trial randomized individuals to one of three 10-session cognitive interventions designed to improve memory, reasoning, or processing speed performance or to a no-contact control condition [21]. Following training there was an immediate posttest and follow-up assessments at 1-, 2-, 3-, and 5-years after intervention. The current study sought to examine only the immediate pretest-posttest data, focusing on the proximal outcome measures used. The initial clinical trial enrolled participants and collected baseline data between 1997 and 2000. Information on potential covariates was collected, including demographic variables, MMSE, recent depressive symptoms, and general health. Testers at all six sites were trained in standardized assessment protocols and quality control by both study investigators, and the coordinating center ensured fidelity to testing procedures.

### 2.2. Participants

The initial sample of 2,802 participants was recruited through on-site presentations, letters to interested persons, newspaper advertisements, introductory letters, and follow-up telephone calls. Participants were cognitively healthy, community-dwelling older adults aged 65 to 94 years (mean age = 73.6 years, mean years of education = 13.5, mean MMSE = 27, 75.8% female, 72% Caucasian, 26% African American, and 2% other minority groups). The majority of participants (64%) were not married, and most reported good to excellent health (84.3%). Efforts were aimed at recruiting older adults independent of care at enrollment, as well as recruiting a diverse sample especially inclusive of African Americans, who were previously under-represented in most cognitive training research. Exclusion criteria included (a) being under age 65 at the start of the study, (b) significant functional and/or cognitive decline at enrollment (e.g., impaired activities of daily living, MMSE < 23, and diagnosis of Alzheimer's disease), (c) having a medical condition disposing the participant to imminent cognitive decline or to mortality within the next 2 years, (d) severe sensory or communicative difficulties precluding participation in assessments and training, (e) having had cognitive training, or (f) planning to be unavailable during the testing and training periods of the study. 

### 2.3. Relationship of the Present Study to the Parent Study

ACTIVE included multiple assessments of participants, but the present paper restricted itself to baseline cognitive performance and the immediate post-test. Neighborhood-level socioeconomic data from publicly available geographic datasets were then merged with ACTIVE data at the individual level. ACTIVE did not aim *a priori *to include multiple measures of individual-level socioeconomic position. Education (highest level achieved) was assessed for all participants, but other person-level socioeconomic indices (e.g., income, occupation) were not collected at baseline; occupational status was collected at the second annual follow-up occasion. 

### 2.4. ACTIVE Cognitive Data

The cognitive domains measured in ACTVE included memory, inductive reasoning, processing speed, everyday cognition, and vocabulary; descriptive statistics on the sample performance for measures within each domain are illustrated in Table 1. Memory was measured using the Hopkins Verbal Learning Test (HVLT, [22]), a 12-word list memory task assessing immediate and delayed recall over 3 learning trials, the Rey Auditory Verbal Learning Test (AVLT, [23]), a 15-word list memory task assessing immediate and delayed recall over 5 consecutive learning trials, and the Rivermead Behavioral Memory Test [24], a paragraph recall task assessing immediate verbal episodic memory. Reasoning was measured using letter series [25], a task requiring accurate identification of the next logical letter group in a series of letters (e.g., a m b a n b a o b), letter sets [26], an inductive reasoning task requiring participants to identify which of 4 sets of letters is unrelated to the others (e.g., eef hhi llm ysy), and word series [27], a task requiring accurate identification of the next logical word in a series. Processing speed was measured using the useful field of view test (UFOV, [28]), a computer-administered task measuring visual sustained, selective, and divided attention through four subtasks, and the complex reaction time test [29], a computer-administered task measuring the time taken to perform various motor behaviors and to complete each task. Everyday cognition was measured using the Everyday Problems Test [30], a performance-based test of ability to solve everyday problems including medications, nutrition, phone use, shopping, and management of finances and household, the Observed Tasks of Daily Living (OTDL) [31], a timed task of problem solving in medication use, telephone use, and financial management, and the Timed Instrumental Activities of Daily Living (TIADL) [32], a timed test of ability to complete daily living tasks (e.g, find telephone number, make change, find and read ingredients on a can, find food items on a shelf, read instructions on medicine bottle). Vocabulary was measured using a multiple-choice measure of vocabulary attainment in which the participant is presented with a word and must choose one of four possible synonyms [26].

### 2.5. Combining ACTIVE and Census Data

Geocoding [33] is a process by which a location (e.g., street address) is assigned Cartesian mathematical coordinates, allowing for other levels of geographic information to be linked with that location. GeoLytics [34], a commercial provider of geocoding services and census demographic data, (1) geocoded the ACTIVE participant addresses, and (2) appended to these addresses data from the 2000 U.S. Census and 2002 Economic Census, creating a dataset that could be used to characterize the neighborhood environment. Following recommendations by prior U.S. neighborhood research [35], ACTIVE participant addresses were geocoded and linked with their associated census tract numbers, which allowed for census tract-level data from the 2000 U.S. Census to be appended to the individual-level ACTIVE data. Geocodes were checked for quality assurance. Participants receiving mail by post office boxes were excluded as such addresses cannot be verified to represent the participant's actual place of residence. Addresses with invalid house numbers, street names, and ZIP codes were flagged for followup; invalid addresses or poorly matched addresses were dropped from the analysis (93% of the ACTIVE addresses accurately matched to a US Geological Survey (USGS) geocode; final sample size = 2,521). 

Census tracts are subdivisions of a county, with an average size of approximately 4,000 residents designed to capture collectively agreed-upon areas approximating neighborhoods. That is, the boundaries of census tracts were agreed upon by local officials knowledgeable of the area and were intended to be homogenous with respect to population characteristics, economic status, and living conditions [35]. Smaller measurement units of area-level SES (e.g., block group) have been shown to correlate highly with census-level measures of SES, and variability in SES among block groups is small relative to variability within their census tract [35]. The association of area-level SES with the cognitive measures was tested at three different levels of area measurement (block group, census tract, and ZIP code), with no significant difference in the strength of associations across levels. Because the census tract has traditionally been the most frequently used and most strongly recommended unit of measurement for area-level effects in health research [36], especially in terms of SEP [3, 35], and because it differed very little in model fit or regression coefficients from the other geographic levels, the census tract level of aggregation was selected for all final analyses involving SEP.

Neighborhood socioeconomic position was measured by creating a socioeconomic position (SEP) index [37]. Because SES variables such as income, education, and occupation are often strongly correlated and have been found to load onto a common factor at the census level [38], census-level data on these variables were combined into a weighted factor score to create a neighborhood SEP index that parsimoniously represented multiple socioeconomic variables. 

### 2.6. Statistical Analysis

The analytical framework for the study involved exploratory and confirmatory factor analysis, structural equation modeling, and repeated measures mixed effects modeling. In keeping with “best practice” in conducting neighborhood research [38], individual- and area-level effects would ideally have been examined using a multi-level modeling (MLM) approach, with samples of individuals clustered in neighborhoods allowing formal assessment of random variation within neighborhoods. However because ACTIVE was not originally designed to sample data stratified by neighborhood but sampled widely across each region, the insufficient sample size within each neighborhood measured precluded the use of MLM. In keeping with recent scholarship, the current study can instead be classified as ecologic, in which neighborhood variables are measured for each person [39]. Statistical Package for the Social Sciences (SPSS) 18.0 and Analysis of Moment Structures (AMOS) 18.0 were used to conduct all statistical analyses. Prior to analysis, all variables were inspected for consistency with the assumption of multivariate normality (to permit maximum likelihood estimation). Where that assumption was not met, Blom transformations [40] were applied to those variables to improve the normality of their distributions. While no data were missing for any neighborhood-level variables, in a few instances data were missing from the ACTIVE dataset's cognitive variables (e.g., due to failure to complete a task or attrition between the baseline and posttraining testing occasions). Models were estimated using full information maximum likelihood (FIML), which uses all available data (thereby not eliminating participants with incomplete data). 

Preparatory to examining the relationship of neighborhood SEP to ACTIVE cognitive outcomes, factor analytic constructs representing neighborhood SEP and baseline cognition were developed. Criteria for acceptable model fit indices included root mean square error of approximation (RMSEA) <0.06, comparative fit index (CFI) >0.95, normed fit index (NFI) >0.95, and Tucker-Lewis Index (TLI) >0.95 [41]. Exploratory factor analyses were first conducted on the neighborhood SEP variables, using Promax rotation to allow optimal fit to the data. A weighted composite measure of SEP was then estimated and optimized in AMOS based on a common factor identified in the exploratory factor analysis. The cognitive measurement model for the present study dimensionalized cognition into several domains, for which the ACTIVE dataset was well designed, having captured at least three measures each of specific cognitive domains including memory, reasoning, processing speed, and everyday cognition, or measures of cognition related to everyday abilities. ACTIVE also collected a measure of vocabulary.

As stated in the introduction, cognitive aging research has increasingly emphasized conceptualization of cognition as both a general ability (“*g*”), and a multifaceted system of specific cognitive domains which may respond differently to neighborhood effects. Therefore the model also estimated effects on each of the above cognitive domains, as well as effects on a higher-order factor representing general cognition or *“g”,* which captures the shared variance among the five cognitive domains in the measurement model. The SEP and cognition factor models were combined in a structural equation model estimating regression paths from SEP to the each of the cognitive factors. Model covariates included age, quadratic age, years of education, gender, and race (White, African American). Measures of individual wealth and duration of residence in neighborhood were not collected in ACTIVE, and could not be included as covariates.

Neighborhood SEP as a predictor of training gains was estimated as repeated-measures mixed effects models [42] rather than as structural equation models because this provided a more flexible interface for the analysis of training gains, which was operationalized as a two-occasion difference score (and thus would not significantly benefit from a latent variable approach) while still permitting FIML estimation for maximum power in the presence of missing data (i.e., using all available cases). Model covariates included age, years of education, gender, and race. Separate models were estimated for each domain trained (memory, reasoning, or speed) to examine the gains of each training group relative to other participants who did not receive that training (i.e., the other two training groups and the no-contact controls). Each model tested SEP as a continuous predictor and occasion (baseline, posttest) and training group (received training on the ability being examined or not) each as class variables. Each model also tested all possible two-way interactions and the three-way interaction of SEP, occasion, and group. Interactions were estimated as residualized interaction terms to correct for collinearity with the model's main effects.

## 3. Results

Neighborhood SEP was initially developed using exploratory factor analysis (EFA) to combine 8 indicators of area-level SES: median household income, % households with income ≥$150,000, % persons in poverty, % with ≤9th grade education, % with ≤ high school education, % with ≥ Bachelor's degree, % unemployed, and % working in management or higher. The EFA indicated all 8 variables loaded strongly onto one factor (eigenvalue = 5.97), explaining 71% of the variance (factor loadings ranged from 0.71 – 0.94). All indicators were then included in a confirmatory factor analysis, where modification indices (MI) suggested high intercorrelations of error variances among variables (e.g., ranging up to MI = 1325.42). To derive an optimally-fit, parsimonious set of variables capturing the shared variance of characteristics on which SEP is typically based (e.g., income, education, and occupational attainment), redundant variables were progressively removed from the model. 

**Figure 1:**
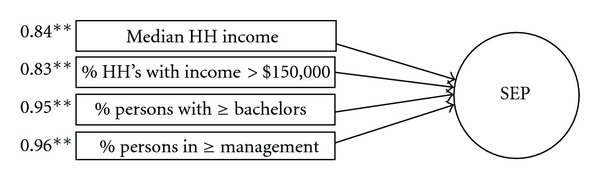
SEP factor structure model. Standardized loadings (ß) to the left of each indicator; **p < 0.001; HH = household.

The final SEP factor model (Figure 1) consisted of four variables measuring socioeconomic advantage in income, education, and occupation (median household income, % with income ≥ $150,000, % with ≥ Bachelor's degree, and % in management or higher). As two indices of area-level income would be expected to correlate, a correlation path was estimated between the error variances of median household income and % income ≥ $150,000 and was retained in all subsequent models. Model fit was good (CFI = 0.999, NFI = 0.999, TLI = 0.996, *χ* ^2^/*df *ratio = 8.23 (*p* = 0.004), and RMSEA = 0.05 (*p* = 0.36)). Notably, the variables loading on the latent SEP factor are all indicators of neighborhood affluence or advantage rather than disadvantage; prior neighborhood research has suggested that measures of social advantage, compared to social disadvantage, may be especially important protective factors in neighborhood influences on development [2]. Mean SEP factor scores were found to differ across ACTIVE catchment sites (*F*(5,2515) = 184.30, *p* < .001; Table 2). Reestimating the models with catchment site as an additional covariate did not alter the pattern of effects, but reduced the fit of the model to an unacceptable extent. Thus, ACTIVE catchment site was tested but not retained in the study models as a covariate.

### 3.1. Measurement Model of Baseline Cognition

**Figure 2:**
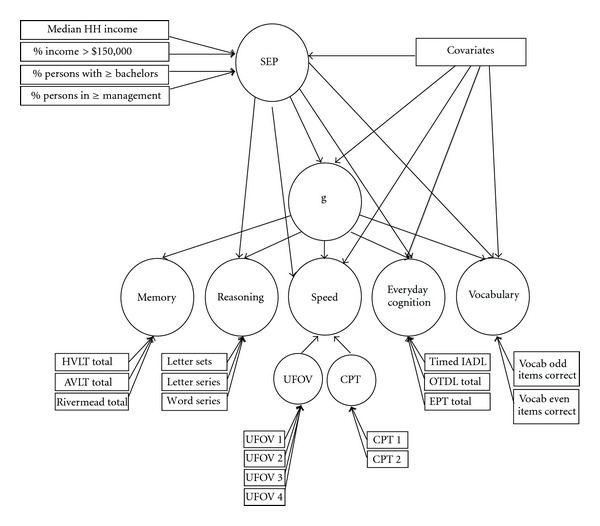
Schematic of the predictive model of baseline cognition. Regression paths were estimated from all covariates (gender, age, education, and race) to g, reasoning, speed, everyday cognition, and vocabulary, and could not be estimated for memory. Model covariates included age, quadratic age, education, gender, and race. HH = Household.

The present paper sought to characterize both general and specific domains of late life cognition. The measured variables (Figure 2) related to memory were combined to represent a memory factor; measures of reasoning combined to form a reasoning factor; guided by preliminary exploratory analyses, measures of processing speed optimally first combined into separate factors based on test, which were then combined to form a speed factor, and individual measures of everyday cognition combined on a factor representing everyday cognition. Because these cognitive domains were represented by latent factors (making them “purer” representations of their domains), the vocabulary measure was also represented as a factor by using odd and even scores as indicators loading on a single vocabulary factor; this effectively transforms that factor into a construct representing the odd-even split half reliable variance of the measure. Model covariates included linear age, the quadratic effect of age, years of education, gender, and race. The disturbance terms of all cognitive factors were permitted to correlate with one another. The error variances of the UFOV 2 and 4 subtests, and the Everyday Problems Test and vocabulary were also allowed to correlate in the process of optimizing model fit (Table 3). As the first-order cognitive factors all correlated with one another (*p* < 0.001), and as discussed earlier, the model included a second-order factor, “*g”*, capturing the variance shared by the cognitive factors (Table 4). Model fit was acceptable (CFI = 0.98, NFI = 0.97, RFI = 0.96, TLI = 0.97, RMSEA = 0.046, (*p *= 1.00), *χ* ^2^/*df* ratio = 6.40, and (*p <* 0.001)). 

### 3.2. Neighborhood SEP Predicting Baseline Cognitive Level

The SEP and cognitive measurement models were combined in a structural equation model (Figure 2) estimating regression paths from neighborhood-level SEP to the cognitive constructs of “*g”*, reasoning, speed, everyday cognition, and vocabulary (a path could not be estimated for SEP or any covariates to memory). Model covariates included linear age, the quadratic effect of age, years of education, gender, and race. Model fit was adequate (CFI = 0.96, NFI = 0.96, TLI = 0.95, *χ* ^2^/*df* ratio = 6.52, (*p* < 0.001), and RMSEA = 0.05 (*p* = 0.99)). After controlling for individual-level predictors, SEP remained a significant predictor of vocabulary alone (*p* < 0.01; Table 5).

### 3.3. Response to Cognitive Training

Repeated-measures mixed effects analyses of variance were used to estimate whether neighborhood SEP predicted differences in training-related gains, beyond practice-related gains, on posttraining measures of memory, reasoning, and speed. Three separate models examined the gains of each training group relative to all other participants (control group plus members of other training groups) who did not receive that particular training (i.e., for the reasoning outcome, the reasoning training group was compared to the control plus memory plus speed training groups). Each model tested SEP as a continuous predictor and occasion (baseline, posttest) and training group (received training on the ability being examined or not), all possible two-way interactions, and the three-way interaction of SEP, occasion, and group. Significant effects for occasion, training group, and their interaction were observed as previously reported in the parent study. A main effect was found for SEP, but for SEP to predict differences in response to ACTIVE training the three-way interaction would have to be significant; this was not found for any of the trained abilities (Memory:  *F*(1, 4594) = 0.66, *p* = 0.42; Reasoning: *F*(1, 4847) = 0.001, *p* = 0.98; Speed: *F*(1, 4793) = 0.001, *p* = 0.97).

## 4. Discussion

The present study sought to examine the relationship between current neighborhood-level socioeconomic position (SEP) and cognitive level, including several specific cognitive domains in the ACTIVE study, and response to cognitive training. The findings demonstrated that, after controlling for individual-level demographic predictors, census-defined neighborhood socioeconomic position independently predicted differences in late life vocabulary as measured in ACTIVE, but not differences in general cognition (“*g”*), reasoning, everyday cognition, or speed. These findings differed from the similar prior study by Brandt [22], which found neighborhood SES effects on a composite cognitive measure capturing cognitive status, verbal fluency, verbal learning, and prospective memory. The results also demonstrated that neighborhood SEP does not predict individual differences in the immediate response to cognitive training in memory, reasoning, or processing speed.

The lack of effect for neighborhood SEP on any fluid cognitive ability (memory, reasoning, speed, and everyday cognition) or on cognitive plasticity (i.e., response to training) is somewhat surprising given the extensive research documenting neighborhood effects on factors affecting brain health, such as cardiovascular fitness and chronic diseases (e.g., [43, 44]). It would be reasonable to hypothesize that neighborhood could indirectly affect fluid cognitive abilities and training gains, which are more sensitive to compromised brain health than vocabulary [45], through differences in access to health care, nutrition, and opportunities for exercise; however, results suggest that if these indirect effects are present they may be relatively weak. Given the relatively good health of this cohort at baseline testing, the majority of respondents reported “good” or “excellent” health, and all were independent of care, there also may not have been sufficient variability in baseline fluid cognition scores for neighborhood effects to be detectable.

The specific association of neighborhood SEP with vocabulary suggests neighborhood influences cognition more through sociocultural mechanisms, as vocabulary captures crystallized cognitive abilities, the dimension of cognition related to stored verbal knowledge, developed over a lifetime of engaging with culture [20]. Vocabulary is accumulated socially through acculturation. Crystallized knowledge is also the only domain that continues to improve in the presence of advancing age, compared to fluid cognitive abilities which become less efficient with age [46]. A review of vocabulary in aging [20] reported increasing vocabulary scores with advancing age, as well as with higher education (although in the present study neighborhood SEP predicted vocabulary independent of education). Vocabulary measures like the one used in this study, requiring multiple-choice word recognition rather than production of word meanings, are especially robust to the effects of aging [20]. 

Neighborhood may influence vocabulary by facilitating or constraining one's capacity for, and influencing the results of, sociocultural engagement in the community. It may do so by providing resources or facilities [47] encouraging cultural interaction or by encouraging acculturation through modeling and social comparisons. That is, high-SEP neighborhoods may support vocabulary because more neighbors with high educational and occupational attainment provide more social models of high achievement. This modeling may foster upward social comparisons [48, 49], pressuring or evoking desire in an individual to be more like his or her neighbors, resulting in greater engagement in activities enhancing cognitive skills and abilities. Positive community social processes may also foster sociocultural interaction. Certainly, other researchers have hinted toward social processes (i.e., social norms, interactions and ties, and collective efficacy) as the mechanism linking neighborhoods with developmental outcomes [2, 47].

While vocabulary is related to current neighborhood SEP, it is important to consider that vocabulary is also highly correlated with childhood cognitive level [45]. Vocabulary often reflects both cognitive reserve and premorbid ability level [50], as it is most robust to not only aging but physical insults to the brain, including head trauma, medical conditions, and exposure to neurotoxins [45]. Expressed differently, when cognition is measured in late life, vocabulary is the strongest index of early life cognitive ability [51]. Therefore, the current neighborhood-vocabulary association found in this study may reflect both historical and current relationships between neighborhood context and cognition; the well-established relationship between neighborhood and cognition in childhood supports this hypothesis. Thus, a part of the late life neighborhood effects on cognition observed here may represent an “echo” of this earlier relationship during a critical period of development. There is evidence that early life socioeconomic indicators also predict late life cognitive outcomes. Berkman and Glymour [52] found that the county of residence during primary schooling, by way of the laws guiding educational requirements in that county, predicted individual differences in both educational attainment and late life cognitive performance. Similarly, Wilson and colleagues [53] reported that parental education during childhood independently predicted cognitive activity across the lifespan and into old age. Therefore, it is likely that childhood SEP, had it been collected in ACTIVE, may be associated with both late life vocabulary and late life neighborhood SEP.

Finally, it was reported in the results that there were catchment site-level differences in mean SEP. Clearly, an individual's neighborhood SEP is also part of the general SEP of the region in which that individual lived; the interaction between neighborhood and regional SEP may influence how a neighborhood's SEP relates to cognition. For example, in regions with lower overall SEP, it may be more “normal” to live in a lower-SEP neighborhood; this experience might differ from the experience of living in a low-SEP neighborhood within a high-SEP region, and might differently affect cognition. Such issues have been explored on the level of individual SES-to-neighborhood SES interactions (e.g., low individual education predicts worse cognition for those living in low-education neighborhoods versus high-education neighborhoods, [13]), but neighborhood-region SEP interactions affecting cognition have not been described to our knowledge. 

### 4.1. Study Limitations and Future Directions

This study was limited in several ways due to its nature as a secondary analysis. Several covariates would ideally have been included had they been collected in the parent study, including individual-level income and occupation (although this may later be examined in for the subset present at the 2nd-annual followup) and the length of time lived at current residence. As a consequence, this study included only a single measure of individual-level socioeconomic status. This is an important limitation, as the observed neighborhood association may be attributable, partially or completely, to unmeasured differences in individual-level socioeconomic status. The possibility that the relationship between vocabulary and neighborhood SEP can be explained by childhood neighborhood SEP must also remain speculation, as this data was not originally collected in ACTIVE. This study therefore was unable to examine measures of childhood socioeconomic status (e.g., mother's or father's education and income, neighborhood SEP in childhood). It is likely that childhood SEP is associated with both vocabulary and late life SEP, in which case vocabulary's relationship with current neighborhood SEP would be expected to diminish or disappear if childhood SEP was accounted for in the study. 

The cross-sectional nature of effects also limits discussion to relationships among variables at a particular time rather than to causal or temporal relationships between variables. Longitudinal assessment of these relationships is an important next step that will be attempted in future studies. At the time this study was conducted, ACTIVE collected its 10th annual follow-up testing occasion, allowing examination of the interaction between 2000 neighborhood and cognition with 2010 neighborhood and cognition. Future studies will also attempt to examine (a) neighborhood effects on change trajectories, and (b) whether there were neighborhood moves for participants, and whether such moves were associated with functional changes. Furthermore, at this follow-up data was collected documenting participant's county of primary schooling. National historical data will allow investigators to use county information to explore whether and how distal environmental influences (i.e., county-level SEP and educational laws during primary schooling years) might predict contextual neighborhood characteristics and cognitive outcomes later in life. 

## 5. Conclusions

This paper adds to the body of research examining neighborhood-cognition associations in late life, and extends previous findings by looking beyond general cognition or cognitive status to examine effects of neighborhood across specific cognitive domains. The finding that neighborhood SEP predicts crystallized cognitive abilities (specifically, vocabulary) suggests that neighborhood effects may be most related to sociocultural influences on cognitive development. There was a lack of association between neighborhood SEP and fluid cognitive abilities, as well as between neighborhood SEP and immediate cognitive change following training. As discussed above, future research should investigate how associations between early life neighborhood context and cognitive development may influence cognitive function and neighborhood selection in late life.

## Figures and Tables

**Table 1:** Minimum, maximum, and mean scores (SD: standard deviation) for the ACTIVE sample on measures of baseline cognition.

Measure	Minimum	Maximum	Mean	SD
HVLT total	4	36	26.05	5.50
AVLT total	0	73	48.45	10.63
Rivermead total	0	17.0	6.30	2.76
Word series total correct	0	30	9.45	4.91
Letter series total correct	0	30	9.99	5.55
Letter sets total correct	0	15	5.72	2.80
UFOV task 1	16	500	30.99	40.71
UFOV task 2	16	500	132.86	124.61
UFOV task 3	43	500	321.09	134.12
UFOV task 4	170	500	456.46	68.64
CRT score 1	0.81	17.0	1.85	0.81
CRT score 2	0.91	18.75	2.25	0.87
OTDL total	1	28	17.58	4.34
EPT total	0	28	18.62	5.76
Vocabulary total correct	0	18	12.37	3.95

**Table 2:** Mean, (standard deviation), and range of SEP indicators and factor scores for the overall study sample and by catchment site.

Site	Median household income	% of households with income >$150,000	% with bachelor degree or higher	% in management positions or higher	SEP factor score
All	44,680.96 (22,448.78) 7,610–170,790	5.7 (8.44) 0–54.0	28.45 (21.48) 1.0–90.0	36.19 (16.15) 4.0–84.0	2.91 (0.93) 0.28–5.76
UAB (*N* = 435)	51,900 (21,214) 7,610–143,968	7.97 (9.18) 0–49.0	37.74 (21.85) 1.0–81.0	42.61 (15.35) 13.0–69.0	3.31 (0.81) 1.04–5.18
IU (*N* = 450)	45,630 (18,390) 12,154–133,479	5.03 (6.33) 0–42.0	30.6 (19.26) 1.0–75.0	36.18 (14.93) 4.0–69.0	2.94 (0.91) 0.36–5.14
HRCA (*N* = 346)	62,359.64 (23,438.77) 18,917–148,257	11.46 (10.54) 0–50.0	48.59 (21.93) 5.0–9.0	52.13 (15.49) 6.0–84.0	3.78 (0.74) 0.93–5.76
JHU (*N* = 415)	32,791.54 (10,699.90) 10,408–84,832	2.0 (2.89) 0–25.0	17.39 (12.38) 2.0–75.0	29.35 (10.4) 10.0–75.0	2.46 (0.64) 0.95–4.59
WSU (*N* = 459)	48,034.58 (28,051.51) 9,615–170,790	7.28 (10.77) 0–54.0	26.42 (21.27) 1–75.0	34.93 (17.83) 7.0–76.0	2.83 (1.03) 0.28–5.41
PSU (*N* = 416)	29,345.72 (8,620.85) 10,101–53,096	1.17 (1.01) 0–6.0	12.72 (6.30) 3.0–28.0	24.30 (8.01) 15.0–44.0	2.27 (0.51) 1.35–3.29

Note: UAB: University of Alabama; IU: Indiana University; HRCA: Hebrew Rehabilitation Centre for Aged; JHU: Johns Hopkins University; WSU: Wayne State University; PSU: Pennsylvania State University.

**Table 3:** Standardized loadings (*β*) of cognitive indicators on first-order cognitive factors. ^∗∗^ *p* < 0.001*. *

Indicator variable	Interim factor	Cognitive factor^∗^	*β*
UFOV1	UFOV		0.53^∗∗^
UFOV2	UFOV		0.78^∗∗^
UFOV3	UFOV		0.80^∗∗^
UFOV4	UFOV		0.65^∗∗^
CRT1	CRT		0.93^∗∗^
CRT2	CRT		0.91^∗∗^
	*UFOV*	Speed	0.77^∗∗^
	*CRT*	Speed	0.77^∗∗^
AVLT total recall		Memory	0.78^∗∗^
HVLT total recall		Memory	0.82^∗∗^
Rivermead total recall		Memory	0.62^∗∗^
Letter series score		Reasoning	0.92^∗∗^
Letter sets score		Reasoning	0.70^∗∗^
Word series score		Reasoning	0.90^∗∗^
Everyday problems test		Everyday cognition	0.83^∗∗^
Timed Instrumental Activities of Daily Living (reverse-coded)		Everyday cognition	0.68^∗∗^
Observed Tasks of Daily Living		Everyday cognition	0.69^∗∗^
Vocabulary, odd items		Vocabulary	0.83^∗∗^
Vocabulary, even items		Vocabulary	0.87^∗∗^

Note: Speed is defined as a second-order factor since better fit was yielded when local associations among UFOV and CRT measures were captured in a first-order factor. These first-order factors were then allowed to load on a second-order speed factor. SeeSection 2for full names of tests used.

**Table 4:** Standardized coefficients (*β*) for loadings of domain-specific cognitive factors on “*g*”. ^∗∗^ *p* < 0.01*. *

Cognitive construct	*β*
Reasoning	0.91^∗∗^
Speed	−0.85^∗∗^
Memory	0.79^∗∗^
Everyday	0.96^∗∗^
Vocabulary	0.64^∗∗^

**Table 5:** Standardized coefficients (*β*) for prediction of cognitive domains by SEP and covariates. ^∗∗^ *p* < 0.01.

			Cognitive factors		
	“*g*”	Speed	Reasoning	Everyday cognition	Vocabulary
SEP	0.02	0.02	0.03	−0.02	0.07^∗∗^
Education	0.37^∗∗^	0.11^∗∗^	0.03	0.09^∗∗^	0.21^∗∗^
Age	−0.49^∗∗^	0.14^∗∗^	0.11^∗∗^	0.13^∗∗^	0.36^∗∗^
Gender	0.34^∗∗^	0.40^∗∗^	−0.27^∗∗^	−0.26^∗∗^	−0.15^∗∗^
Race	0.35^∗∗^	0.02	0.01	0.02	0.13^∗∗^

Note: Beta weights to general cognition, or *g*, are predicting total variance in that factor. Beta weights to the remaining cognitive factors represent additional significant predictor effects after controlling for *g*.
